# Mucin-producing tumors of the ovary——preoperative differentiation between metastatic ovarian mucinous carcinoma and primary mucinous malignant tumors

**DOI:** 10.1186/s13048-024-01382-8

**Published:** 2024-03-13

**Authors:** Song-Qi Cai, Min-Rong Wu, Xiao-Liang Ma, Jing-Jing Lu, Jin-Wei Qiang, Yin-Yin Guan, Meng-Su Zeng, Jian-Jun Zhou

**Affiliations:** 1 Department of Radiology, Zhongshan Hospital, Shanghai Medical College, Fudan University, Xvhui District, Shanghai, PR China; 2 Shanghai Institute of Medical Imaging, Xvhui District, Shanghai, PR China; 3 Department of Cancer Center, Zhongshan Hospital, Fudan University, Xvhui District, Shanghai, PR China; 4 Department of Radiology, Zhongshan Hospital Fudan University Xiamen Branch, Jinhu Road No.668, Huli District, 361015 Xiamen, Fujian PR China; 5 Xiamen Municipal Clinical Research Center for Medical Imaging, Xiamen, China; 6 Xiamen Key Clinical Specialty for Radiology, Xiamen, China; 7 Department of Radiology, Jinshan Hospital, Shanghai Medical College, Fudan University, Jinshan District, Shanghai, PR China; 8 Department of Pathology, Zhongshan Hospital (Xiamen), Fudan University, Xiamen, PR China

**Keywords:** Ovarian mucinous carcinoma, Primary, Metastatic, Magnetic resonance imaging

## Abstract

**Objective:**

To investigate the clinical and magnetic resonance imaging (MRI) features for preoperatively discriminating  primary ovarian mucinous malignant tumors (POMTs) and metastatic mucinous carcinomas involving the ovary (MOMCs).

**Methods:**

This retrospective multicenter study enrolled 61 patients with 22 POMTs and 49 MOMCs, which were pathologically proved between November 2014 to Jane 2023. The clinical and MRI features were evaluated and compared between POMTs and MOMCs. ﻿Univariate and multivariate analyses were performed to identify the significant variables between the two groups, which were then incorporated into a predictive nomogram, and ROC curve analysis was subsequently carried out to evaluate diagnostic performance.

**Results:**

35.9% patients with MOMCs were discovered synchronously with the primary carcinomas; 25.6% patients with MOMCs were bilateral, and all of the patients with POMTs were unilateral. The biomarker CEA was significantly different between the two groups (*p* = 0.002). There were significant differences in the following MRI features: tumor size, configuration, enhanced pattern, the number of cysts, honeycomb sign, stained-glass appearance, ascites, size diversity ratio, signal diversity ratio. The locular size diversity ratio (*p* = 0.005, OR = 1.31), and signal intensity diversity ratio (*p* = 0.10, OR = 4.01) were independent predictors for MOMCs. ﻿The combination of above independent criteria yielded the largest area under curve of 0.922 with a sensitivity of 82.3% and specificity of 88.9%.

**Conclusions:**

Patients with MOMCs were more commonly bilaterally and having higher levels of CEA, but did not always had a malignant tumor history. For ovarian mucin-producing tumors, the uniform locular sizes and signal intensities were more predict MOMCs.

## Background

﻿Metastases found in the ovaries are common. About 17.4%-30.0% of ovarian malignancies are secondary masses [1–3]. ﻿The most common histological subtype is ﻿mucin-producing tumors, originated from gastrointestinal tract, cervix and ﻿biliary tree, et al. [4, 5].﻿ The prognosis of primary ovarian mucinous malignant tumors (POMTs) and metastatic mucinous carcinomas involving the ovary (MOMCs) is different. The prognosis of MOMCs seems to be associated with the origin of ovarian metastatic carcinoma and optimal debulking surgery [6, 7]. The 5-year survival rates of debulking surgery-treated ovarian mucinous carcinoma originating from gastric, colorectal, and breast tumors are 0%, 20.7% and 22.2%, respectively [8, 9]. The overall prognosis of POMTs is excellent, encompassing both mucinous borderline tumors and carcinomas. The current working hypothesis for the origin of primary ovarian mucinous carcinoma involves a multistep transformation from a borderline mucinous tumor [10]. Mucinous borderline ovarian tumors are the most common borderline tumors, accounting for 70% of such tumors, with an overall survival of 98% at 5 years and 96% at 10 years. ﻿Mucinous ovarian cancers are the least common histological type, comprising 3% of ovarian cancers, with 83% patients at FIGO (International Federation of Gynecology and Obstetrics) stage I and boasting a 91% 5-year survival rate [11–13]. The treatment for MOMCs is tailored to the primary organ site. ﻿The effectiveness of chemotherapy for POMTs is still controversial [14].

The distinction between POMTs and MOMCs is often problematic. In the clinical setting, patients’ past history of malignancy may be helpful in distinguishing primary from metastatic ovarian tumors. Occasionally, the primary tumors of MOMCs may remain clinically silent, only presenting with symptoms related to an ovarian mass, and they may not manifest until a period of time after a total abdominal hysterectomy with bilateral salpingo-oophorectomy. A correct diagnosis is the mainstay of treatment, and methods to preoperatively differentiate between POMTs and MOMCs are needed for insight.

So far, only a few studies have investigated the differences between POMTs and MOMCs [3, 6, 15–18]. Most of them appeared as multilocular cystic masses. However, they found the size and laterality were ﻿the diagnostic approaches for the MOMCs [18]. The frozen section diagnosis of mucinous tumors of the ovary can be quite difficult due to the size and heterogeneity of these tumors [16, 19]. Therefore, effective preoperative diagnostic approaches for POMTs and MOMCs may be helpful in clinical setting and deserve further study.

In this study, we conducted a multicenter study of patients with mucin-producing tumors. We investigated the preoperative clinical data and magnetic resonance imaging (MRI) characteristics of the mucin-producing tumors. The aim was to gain a deep understanding of the differences between MOMCs and POMTs.

## Methods

### Patient population

**Fig.1:**
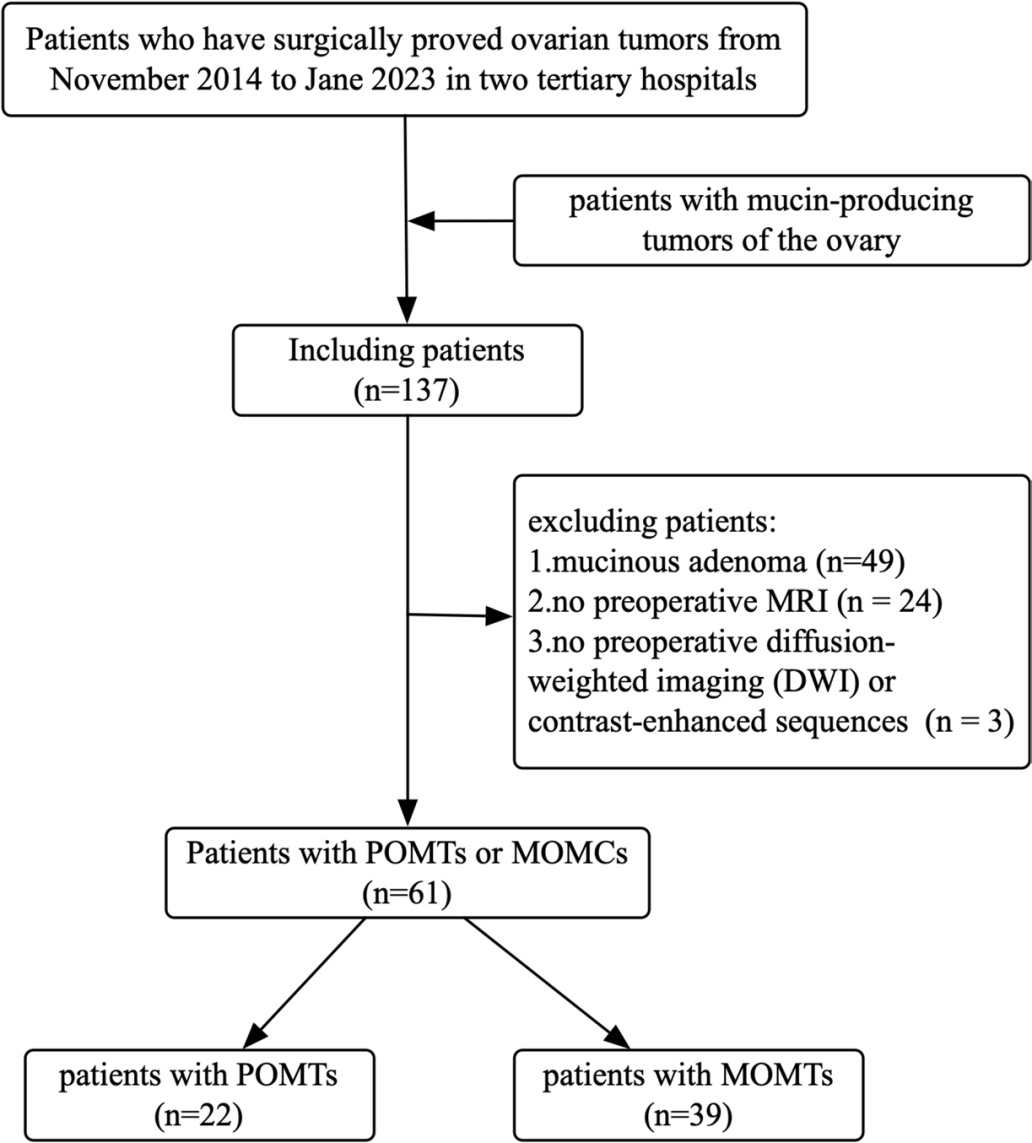
The inclusion and exclusion criteria of patients. POMT, primary ovarian mucinous malignant tumor; MOMC, metastatic ovarian mucinous carcinoma

﻿This retrospective multicenter study received approval from each institutional review board, and informed consent was obtained though an opt-out on the website. The study involved 137 patients with histopathologically proved mucin-producing ovarian tumors, diagnosed using surgical specimens from November 2014 to Jane 2023. ﻿Seventy-six patients were excluded for the following reasons:1) mucinous adenoma (*n* = 49); 2) lack of preoperative MRI (*n* = 24); 3) absence of preoperative diffusion-weighted imaging (DWI) or contrast-enhanced MRI (*n* = 3) (Fig. 1). The remaining 61 patients included 22 patients with POMTs [age range, 31–66; median age 52 (31, 66) years] and 39 patients with MOMCs [age range, 23–79; median age 49 (38, 56) years]. Their clinical data and preoperative MR images were evaluated.

### MRI Protocol

﻿All MRI examinations were conducted on 1.5 T or 3.0 T MR systems (﻿Discovery MR 750, GE, Healthcare; Avanto, MAGNETOM Verio, Siemens Healthcare) using a dedicated phased-array body coil and spine coil. The conventional pelvic MRI protocol included T1-weighted imaging (T1WI) using spin-echo without fat saturation, fat-suppressed T2-weighted imaging (T2WI) with turbo spin-echo, as well as three-phase axial contrast-enhanced T1WI (30, 60, and 150 s acquisitions) using three-dimensional T1WI with fat suppression after the intravenous administration of 0.2 mmol/kg of gadopentetate dimeglumine at a rate of 2 mL/s. An axial DWI (b = 0, 800 or 1000 s/mm^2^) sequence was obtained before contrast-agent injection using a single-shot echo planer imaging sequence: repetition time / echo time = 3200 ms / 87 ms, section thickness = 4–5 mm with-out gap, and the number of excitations = 4. ﻿All other MR images were obtained as follows: slice thickness 3–4 mm; gap 1–2 mm; field of view 200–250 mm × 340 mm. The total scale time was 20-25 min.

### ﻿Image analysis

﻿﻿Two readers (C.S.Q. and W.M.R., with 12 and 5 years of gynecological imaging experience, respectively), who were blinded to the patients’ clinical and histopathologic data, independently reviewed the conventional images on PACS (Pathspeed, GE Medical Systems Integrated Imaging Solutions, Prospect, IL). The following MRI features were evaluated: (i) tumor size: maximum diameter; (ii) configuration: cystic lesion; ﻿﻿lesion with solid tissue, papillary formations, mural nodules, irregular cyst wall or septations; solid lesion, ﻿consists of at least 80% solid tissue with < 20% of lesion volume being cystic; (iii) number of cysts: unicystic; oligocystic, < 10 cysts; multicystic, ≥ 10 cysts; (iv) ﻿honeycomb loculi: ﻿a densely aggregated numerous loculi of 5–10 mm; (v) stained-glass appearance: different signal intensity of each cystic portion within multilocular cystic lesions on T1WI and T2WI; ﻿(vi) ﻿﻿fluid–fluid level: appearance in which the nondependent fluid component has a different signal intensity from the dependent fluid component with horizontal delineation; (vii) ﻿shading sign: ﻿cyst fluid that is hypointense on T2WI (extent of hypointense T2 signal intensity may be homogeneous, variable within the cyst or graduated and dependent) [20]; (viii) size ﻿diversity ratio: ﻿the size ratio of the smallest/largest loculus on contrast T1WI ﻿in oligocystic or multilocular tumors; (ix) ﻿signal intensity diversity ratio: ﻿the most varying signal intensity on T2WI ﻿in oligocystic or multilocular tumors; (x) enhancement of the solid tissue (﻿less than or equal to the myometrium, or ﻿greater than the myometrium ﻿at 30-40 s post injection) [20]; (xi) enhanced pattern (low-risk: signal intensity lower than that of myometrium at 30 s and 60 s; intermediate -risk: signal intensity lower than that of myometrium at 30 s and higher than that at 60 s; high-risk: signal intensity higher than that of myometrium at 30 s); (xii) apparent diffusion coefficient (ADC) values of the solid components; (xiii) ascites, graded as none or physiological (limited to the Douglas pouch), moderate (limited to the pelvic cavity) or massive ascites (beyond the pelvic cavity). Discrepancies on the categorical features were resolved in consensus. The mean quantitative values of the tumor size and ADC value were adopted by averaging the measurements from two radiologists.

### Statistical analysis

All statistical analyses were performed using SPSS software (V26.0; IBM, Armonk, NY) and ﻿R software (V3.4.1; Boston, MA, USA). All tests were two-sided, and *p* < 0.05 was considered significant. Continuous variables with a normal distribution were presented as mean ± standard deviation, while non-normal variables were presented as the median (25th percentile and 75th percentile). Interobserver agreements between the two readers for imaging analysis were assessed using the interclass correlation coefficient (ICC): poor, 0–0.2; fair, 0.2–0.4; moderate, 0.4–0.6; good, 0.6–0.8; excellent, 0.8–1.0. The clinical and MRI findings between the POMTs and MOMCs groups were compared using the independent sample *t*-test or Mann–Whitney *U* test for continuous variables and Pearson’s x^2^ test or Fisher’s exact test for categorical variables. Factors with a *p* value less than 0.05 in univariate analyses were entered into the multivariate models. Multivariate logistic regression analysis was performed using the forward LR elimination method to identify the independent predictors. Receiver operating characteristic (ROC) analyses of both independent and combined significant findings were conducted to assess the ability of the measurements to discriminate between the two groups. The corresponding areas under the ROC curves (AUCs), sensitivities, specificities, and 95% confidence intervals (95% CI) were calculated. A nomogram was built using the predictive model to serve as a graphical representation of the results. The nomogram’s predictive performance was measured using Harrell’s C-index and calibration with 1000 bootstrap samples to decrease the overfit bias [21]. A calibration curve was plotted to analyze the nomogram’s diagnostic performance. The Hosmer–Lemeshow test was used to assess the agreement between the nomogram and true subtype from the calibration curve. Decision curve analysis was carried out to determine the clinical usefulness of the nomogram by quantifying the net benefits under all threshold probabilities.

## Results

### Patient characteristics

﻿A total of 61 patients with mucin-producing tumors were included in this study. Twenty-two patients (8 carcinomas, 14 borderline tumors) with 22 masses were proved to have PMOTs, and 39 patients (23 colons; 8 gastric; 2 appendix; 3 cervix; 2 bile duct; 1 pancreas) with 49 masses had MOMCs. Ten (25.6%) of 39 patients with MOMCs were bilateral, and all patients with POMTs were unilateral. Fourteen (35.9%) of 39 patients with MOMCs and the primary carcinomas were discovered synchronously; 25 (64.1%) of 39 patients were metachronous. There were no significant differences in human epididymis 4 (HE4), ﻿cancer antigen 125 (CA125), and cancer antigen 199 (CA199)﻿ levels between the two groups, except for carcinoembryonic antigen (CEA), which was significantly higher in MOMCs (*p* = 0.002). Baseline patient’s demographic and pathologic characteristics are summarized in Tables 1 and 2.

**Table 1:** Clinical characteristics of patients with mucin-produced tumors

Variables	PMOT	MOMC	*p*
Age(year), median (25th percentile and 75th percentile)	52(31, 66)	49(38, 56)	*0.152*
HE4 (U/mL), median (25th percentile and 75th percentile)	60.6(44.2, 85.0)	57.3(52, 88.8)	*0.717*
CA125(U/mL), median (25th percentile and 75th percentile)	41.6(22.4, 84.5)	30.9(17.3, 67.45)	*0.447*
CA199(U/mL), median (25th percentile and 75th percentile)	27(13.5, 246)	37.7(13.8, 220)	*0.837*
CEA(5U/mL), median (25th percentile and 75th percentile)	1.8(1.3, 2.9)	5.5(2.9, 14.2)	*0.002*
The primary malignant tumor history (Y/N)	22/0	14/25	*0.000*
FIGO	22	39	*-*
I	20	\	
II	0	\	
III	1	\	
IV	1	39	

*POMT* Primary ovarian mucinous malignant tumor, *MOMC* Metastatic ovarian mucinous carcinoma, *HE4* Human epididymis 4, *CA125* Cancer antigen 125, *CA199* Cancer antigen 199, *CEA* carcinoembryonic antigen

**Table 2:** Pathological characteristics of patients with mucin-produced tumors

Variables	PMOT	MOMC	*p*
Location(n, %)	22	39	0.000
Unilatery	22 (100.0%)	29(74.4%)	
Bilatery	0	10(25.6%)	
The primary malignant tumor histopathology subtypes (n, %)	-	39	-
Gastric caner	\	8 (20.5%)	
Colorectal cancer	\	23 (60.0%)	
Endocervical cancer	\	3(7.7%)	
Appendiceal cancer	\	2(5.1%)	
Cholangiocarcinoma	\		
Pancreatic cancer	\	1(2.6%)	
Histopathological grade (n, %)	22	-	-
borderline tumor	14(63.6%)	\	
carcinoma	8(36.4%)	\	

*POMT* Primary ovarian mucinous malignant tumor, *MOMC* Metastatic ovarian mucinous carcinoma

### Imaging characteristics

**Fig.2:**
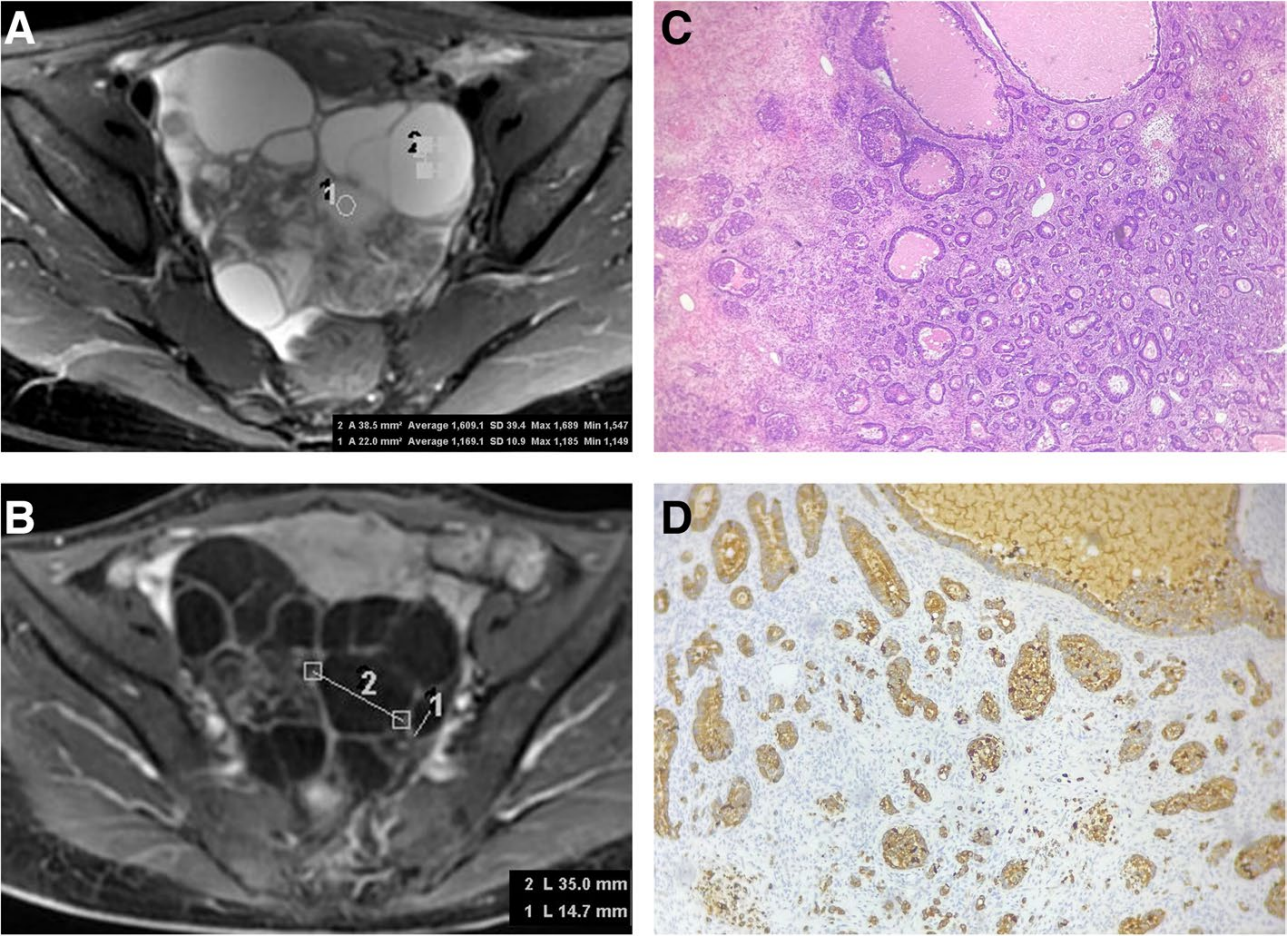
A 42-year-old female patient with bilaterally ovarian metastases from ﻿colorectal adenocarcinoma. ﻿The locular signal intensity is uniform on axial T2-weighted images (**A**). There is varying size between the cystic loculations on axial contrast-enhanced T1-weighted images (**B**). The signal and size diversity ratio are 1.38 and 2.38, respectively. There are numerous microcapsules with a solid portion invasion (**C**, hematoxylin and eosin stain × 100 magnification). The epithelial cells show strong immunoreactivity for cytokeratin 20 (**D**, × 100 magnification)

**Fig.3:**
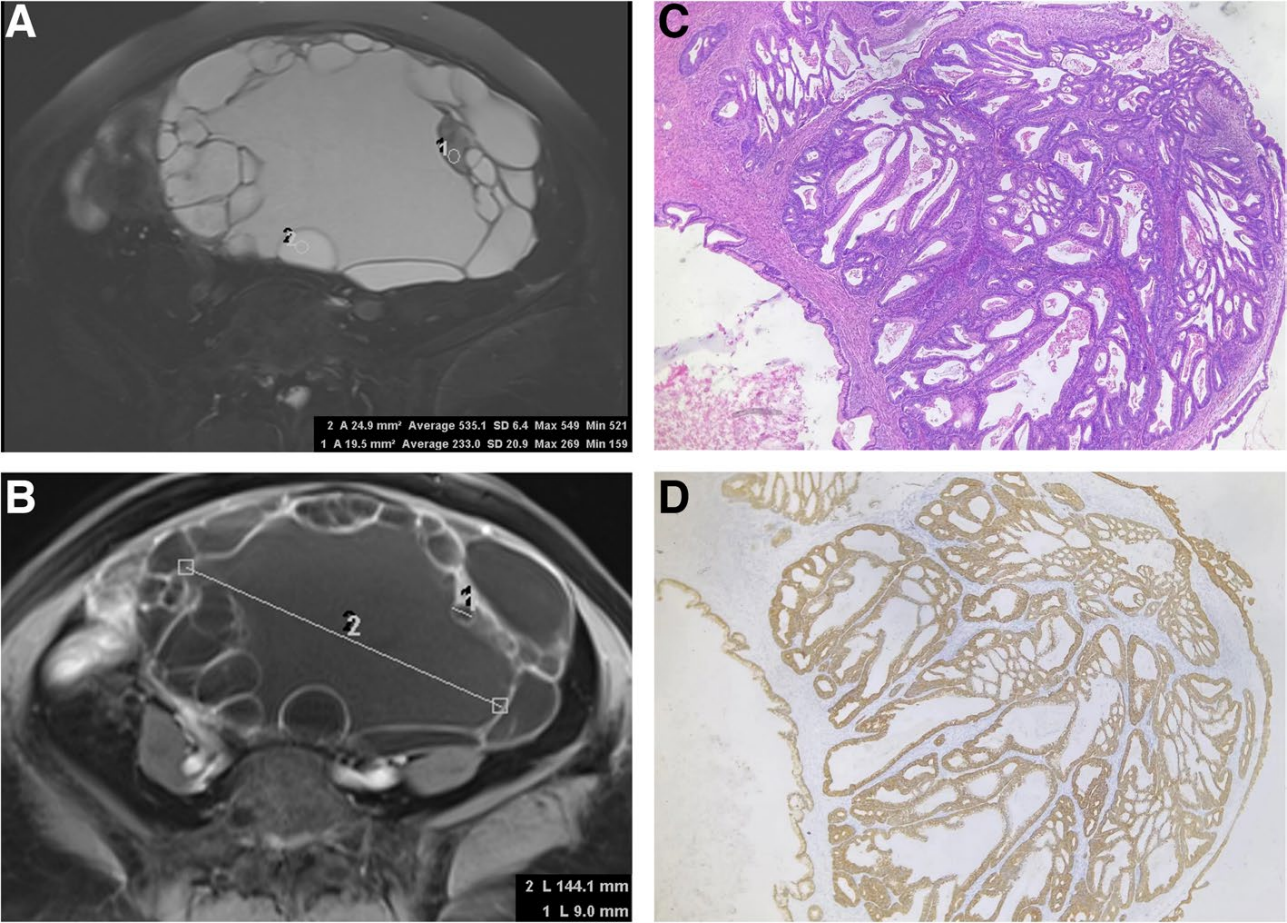
A typical primary ovarian borderline mucinous cystadenoma in a 69-year-old female patient. The ovarian tumor is composed of cysts of varying signal intensity on axial T2-weighted images (**A**) and varying signals on axial contrast-enhanced T1-weighted images (**B**). The signal and size diversity ratio are 2.30 and 16.0, respectively. ﻿Abundant cystic glandular structures with different sizes edged by a multistratified epithelium (**C**, hematoxylin and eosin stain × 100 magnification). The epithelial cells show strong immunoreactivity for cytokeratin 7 (D, × 100 magnification)

﻿All of the imaging characteristics showed good to excellent interobserver agreements. Univariate analysis revealed significant differences in tumor size, configuration, enhanced pattern, the number of cysts, honeycomb sign, stained-glass appearance, size diversity ratio, signal intensity diversity ratio, and ascites between the two groups. Compared with POMTs, MOMCs exhibited more common MRI characteristics: (i) smaller tumor size; (ii) solid lesion; (iii) high-risk enhanced pattern; (iv) moderate ascites; (vi) low locular size and signal intensity diversity ratio (Table 3). MOMCs were less likely to have honeycomb sign and stained-glass appearance (Figs. 2 and 3).

**Table 3:** Comparison the MRI characteristics of mucin-produced tumors between the POMT and MOMC groups

Variables	POMT	MOMC	*p*	ICC(95% CI)
Tumor size	22	49		
Median (25th percentile and 75th percentile)(mm)	137.85(62.875, 209.75)	68.2(48.6, 98.75)	0.001*	0.923(0.814, 0.977)
Configuration	22	49	0.000*	0.868(0.743, 0.926)
Cystic lesion	2(9.1%)	6(12.2%)		
Lesion with solid component	19(86.4%)	19(38.8%)		
Solid lesion	1(4.5%)	24(49.0%)		
Number of cysts	22	49	0.069	0.799(0.666, 0.877)
Unicystic	6(27.3%)	9(18.4%)		
Oliocystic	4(18.2%)	23(46.9%)		
Multicystic	12(54.5%)	17(34.7%)		
Honeycomb sign	22	49	0.023*	0.802(0.699, 0.873)
Yes	11(50.0%)	39(79.6%)		
No	11(50.0%)	10(20.4%)		
Stained-glass appearance	22	49	0.003*	0.818(0.721, 0.883)
Yes	11(50.0%)	40(81.6%)		
No	11(50.0%)	9(18.4%)		
Fluid–fluid level	22	49	0.928	0.853(0.792, 0.907)
Yes	21(95.5%)	47(95.9%)		
No	1(4.5%)	2(4.1%)		
Shading	22	49	0.632	0.767(0.626, 0.855)
Yes	14(63.6%)	34(69.4%)		
No	8(36.4%)	15(30.6%)		
Size diversity ratio	15.04(9.86, 24.58)	5.80(3.31, 7.88)	0.000*	0.807(0.670, 0.886)
Signal intensity diversity ratio	1.72(1.36, 2.74)	1.21(1.09, 1.55)	0.006*	0.823(0.723, 0.903)
Enhancement^a^	22	44	0.460	0.647(0.412, 0.786)
Less than or equal to the myometrium	16(72.7%)	28(63.6%)		
Greater than the myometrium	6(27.3%)	16(36.4%)		
Enhanced pattern^a^	22	44	0.010*	0.839(0.618, 0.920)
Low risk	16(72.7%)	24(54.5%)		
Intermediate risk	6(27.3%)	6(13.6%)		
High risk	0(0.00%)	14(31.8%)		
ADC value	1.35(1.04, 1.60)	1.16(0.98, 1.52)	0.722	0.907(0.852, 0.942)
Ascites	22	49	0.006*	0.892(0.827, 0.933)
None or physiological	10(45.5%)	10(20.4%)		
Moderate	10(45.5%)	39(79.6%)		
Massive	2(9.0%)	0(0.00%)		

*POMT* primary ovarian mucinous malignant tumor, *MOMC* metastatic ovarian mucinous carcinoma

^*^p < 0.05

^a^five patients without obvious solid tissue in MOMC group, *ICC* Interclass correlation coefficient, *CI* confidence interval

﻿Multivariable analysis revealed that the size diversity ratio (*p* = 0.005, OR = 1.31) and signal diversity ratio (*p* = 0.10, OR = 4.01) were significant differentiating MRI features. The optimal cut-off ratios of size and signal diversity were 10 and 1.46, respectively, with sensitivity, specificity, and AUC of 83.0%, 77.8% and 0.856, and 85.1%, 77.8%, and 0.805, respectively (Table 4).

**Table 4:** Diagnostic performances of the independent criteria and their combination for differentiating POMCs from MOMCs

Parameters	AUC	95% CI	Threshold	Sensitivity	Specificity	*p*_*1*_	*p*_*2*_
Size diversity ratio	0.856	0.744, 0.968	10	83.0%	77.8%	/	0.051
Signal intensity diversity ratio	0.805	0.685, 0.925	1.46	85.1%	77.8%	0.051	/
Combination	0.922	0.856, 0.988	0.281	82.3%	88.9%	0.066	0.117

*POMC* Primary ovarian mucinous malignant tumors, *MOMC* Metastatic ovarian mucinous carcinomas, *AUC* Areas under the curve, *CI* Confidence interval, *p*_*1*_ Size diversity ratio compared with other features, *p*_*2*_ signal diversity ratio compared with other features

### Nomogram development

**Fig.4:**
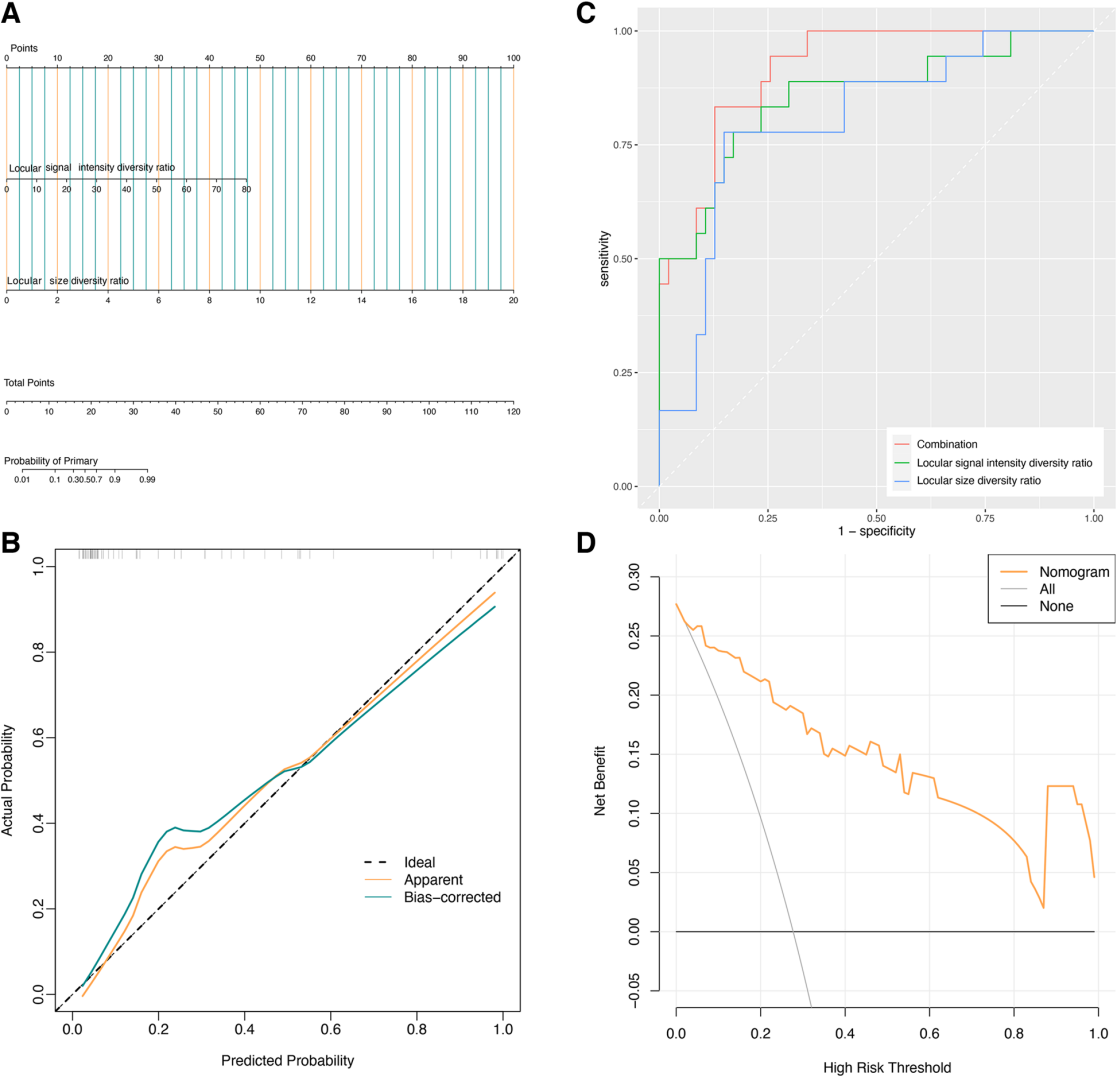
ROC analysis, nomogram, calibration and decision curves for differentiating POMT and MOMC. **A** The nomogram established by combining the locular size diversity ratio and signal intensity diversity ratio. Predictor points are shown on uppermost point scale corresponding to each variable. On the bottom scale, points for all variables were added and translated into POMT positivity probability. **B** The calibration curve depicts the model calibration in terms of the agreement between the predicted probabilities and observed outcomes of POMT. The dotted black line represents an ideal prediction, whereas the solid black line shows the nomogram’s predictive ability. **C** The combination attained the highest receiver characteristic operating curve value. **D** The gray line represents the net benefit of assuming that all patients had MOMC, the black line shows the net benefit of assuming that none of the patients had POMT, and the yellow line resembles the expected net benefit per patient based on the predictive nomogram. POMT, primary ovarian mucinous malignant tumor; MOMC, metastatic ovarian mucinous carcinoma

The nomogram that integrated the size diversity ratio and signal diversity ratio to predict POMTs is presented in Fig. 4A. Satisfactory predictive performance of the nomogram was observed, with a C-index value of 0.915 (95%CI: 0.854–0.983). The calibration curve for the nomogram’s POMTs probability showed good agreement between prediction and observation (*p* < 0.001; Fig. 4B). The ROC curve analysis of the model combined locular size diversity ratio and signal intensity diversity ratio yielded a ﻿largest AUC of 0.922, with a sensitivity of 82.3%, and a specificity of 88.9% for distinguishing POMTs from MOMCs (Fig. 4C). The net benefit of the decision curve was greater than the curve assuming all patients had POMTs (Fig. 4D).

## Discussion

In clinical setting, patients’ past history of malignancy may be helpful in distinguishing primary from metastatic ovarian tumors; however, ﻿35.9% metastatic ovarian tumors are an initial symptom in our cohort. According to previous reports, 56%-75% MOMCs and the primary carcinomas were discovered synchronously; 44% were metachronous [22]. The level of CEA was significantly higher in MOMCs group, which was in accordance with previous studies [23]. Though HE4 was considered specific elevation in epithelial tumor of ovary, there is no significant difference between the POMTs and MOMCs in our cohort.

﻿Seidman et al. advocated an algorithm that could differentiate primary mucinous ovarian neoplasms from metastatic ones by gross inspection in pathology [15]. The simple rule classifies all bilateral mucinous carcinomas as metastatic, unilateral mucinous carcinomas < 10 cm as metastatic, and unilateral mucinous carcinomas ≥ 10 cm as primary. Although bilateral lesions were also more commonly found in MOMC group in our cohort, only the diverse size and signal intensity of the cysts were independent predictors in our cohort. Moreover, other studies found that serous papillary and endometrioid carcinomas, well-known forms of ovarian cancer, often exhibit bilateralism [24].

﻿POMTs are well-known for appearing as multilocular cystic masses with varying signal intensities (stained-glass appearance) [25]. According to clinicopathologic analysis, most Krukenberg tumors of the ovary were typically solid and ranged from firm to edematous to gelatinous, with one-third of the tumors having cysts [26]. Those of colorectal origin often appear as multilocular cystic masses [16, 27]. ﻿The overall morphology varied according to the prominence of signet-ring cells, extracellular mucin, edema, and various epithelial patterns [18]. Only one lesion had no cyst, and 10.2% tumors were cystic lesions without solid tissue in MOMC group.

For lesions with multicysts, the locular diverse size and signal intensity were independent predictors, broadly in accordance with ﻿Yumiko Oishi Tanaka’s results [17]. ﻿It may be due to the more homogeneously proliferate nature of metastatic ones compared to those of primary tumors. As we known, POMTs appear to evolve in a stepwise fashion from benign epithelium to a preinvasive lesion to carcinoma, which means mucinous ovarian cancer is frequently mixed with areas of mucinous cystadenoma or precancerous lesions (borderline mucinous tumor, borderline tumor with intraepithelial carcinoma, microinvasive carcinoma, or a combination of such lesions) [28, 29].

There are some limitations in our study. Firstly, preliminary results may be affected by the retrospective nature of this study. Therefore, prospective randomized trials with a larger sample are warranted to validate the generalization capabilities of the results. Secondly, we did not take into account other histopathology subtypes, such as ﻿struma ovarii, which also have a multilocular cystic appearance. Because of diversity protein contained, the morphology of which is similar to PMOT [30]. Most of these primary neoplasms may be cured by optimal debulking surgery at an early stage. Thirdly, we did not take into consideration ovarian tumors, which often bilateralism. However, most of them have cystic-solid appearance and rarely present as multilocular cystic tumors.

In conclusion, patients with MOMCs were more commonly bilaterally and had higher level of CEA, but not always had malignant tumor history. When encountering a multilocular ovarian mass suspected of being mucin-producing tumors, the possibility of MOMC than POMT should be considered when encountering uniform locular sizes and signal intensities.

## References

[1] Zhang JJ, Cao DY, Yang JX, Shen K (2020). Ovarian metastasis from nongynecologic primary sites: a retrospective analysis of 177 cases and 13-year experience. J Ovarian Res..

[2] Moore RG, Chung M, Granai CO, Gajewski W, Steinhoff MM (2004). Incidence of metastasis to the ovaries from nongenital tract primary tumors. Gynecol Oncol.

[3] Khunamornpong S, Suprasert P, Pojchamarnwiputh S, Na Chiangmai W, Settakorn J, Siriaunkgul S (2006). Primary and metastatic mucinous adenocarcinomas of the ovary: evaluation of the diagnostic approach using tumor size and laterality. Gynecol Oncol.

[4] Zulfiqar M, Koen J, Nougaret S, Bolan C, Vanburen W, McGettigan M (2020). Krukenberg tumors: Update on imaging and clinical features. Am J Roentgenol.

[5] Kelemen LE, Köbel M (2011). Mucinous carcinomas of the ovary and colorectum: different organ, same dilemma. Lancet Oncol.

[6] Heatley MK (2012). Mucinous tumours of the ovary–primary and metastatic. J Clin Pathol.

[7] Karabuk E, Faruk Kose M, Hizli D, Taşkin S, Karadaǧ B, Turan T (2013). Comparison of advanced stage mucinous epithelial ovarian cancer and serous epithelial ovarian cancer with regard to chemosensitivity and survival outcome: a matched case-control study. J Gynecol Oncol.

[8] Simons M, Ezendam N, Bulten J, Nagtegaal I, Massuger L (2015). Survival of patients with mucinous ovarian carcinoma and ovarian metastases: a population-based cancer registry study. Int J Gynecol Cancer.

[9] Jiang R, Tang J, Cheng X, Zang RY (2009). Surgical treatment for patients with different origins of Krukenberg tumors: outcomes and prognostic factors. Eur J Surg Oncol.

[10] Seidman JD, Horkayne-Szakaly I, Haiba M, Boice CR, Kurman RJ, Ronnett BM (2004). The histologic type and stage distribution of ovarian carcinomas of surface epithelial origin. Int J Gynecol Pathol.

[11] Richardson MT, Mysona DP, Klein DA, Mann A, Liao CI, Diver EJ (2020). Long term survival outcomes of stage I mucinous ovarian cancer - A clinical calculator predictive of chemotherapy benefit. Gynecol Oncol.

[12] Khunamornpong S, Settakorn J, Sukpan K, Suprasert P, Siriaunkgul S (2011). Mucinous tumor of low malignant potential (borderline or atypical proliferative tumor) of the ovary: a study of 171 cases with the assessment of intraepithelial carcinoma and microinvasion. Int J Gynecol Pathol.

[13] Prat J (2015). Abridged republication of FIGO’s staging classification for cancer of the ovary, fallopian tube, and peritoneum. Cancer.

[14] Wang F, Yang Y, Du X, Zhu X, Hu Y, Lu C (2023). Claudin18.2 as a potential therapeutic target for primary ovarian mucinous carcinomas and metastatic ovarian mucinous carcinomas from upper gastrointestinal primary tumours. BMC Cancer.

[15] Seidman JD, Kurman RJ, Ronnett BM (2003). Primary and metastatic mucinous adenocarcinomas in the ovaries: incidence in routine practice with a new approach to improve intraoperative diagnosis. Am J Surg Pathol.

[16] Dundr P, Singh N, Nožičková B, Němejcová K, Bártů M, Stružinská I (2021). Primary mucinous ovarian tumors vs. ovarian metastases from gastrointestinal tract, pancreas and biliary tree: a review of current problematics. Diagn Pathol.

[17] Tanaka YO, Okada S, Satoh T, Matsumoto K, Oki A, Saida T (2013). Diversity in size and signal intensity in multilocular cystic ovarian masses: new parameters for distinguishing metastatic from primary mucinous ovarian neoplasms. J Magn Reson Imaging.

[18] Hu J, Khalifa RD, Roma AA, Fadare O (2018). The pathologic distinction of primary and metastatic mucinous tumors involving the ovary: a re-evaluation of algorithms based on gross features. Ann Diagn Pathol.

[19] Storms AA, Sukumvanich P, Monaco SE, Beriwal S, Krivak TC, Olawaiye AB (2012). Mucinous tumors of the ovary: diagnostic challenges at frozen section and clinical implications. Gynecol Oncol.

[20] Reinhold C, Rockall A, Sadowski EA, Siegelman ES, Maturen KE, Vargas HA (2021). Ovarian-adnexal reporting lexicon for MRI: a White Paper of the ACR ovarian-adnexal reporting and Data systems MRI Committee. J Am Coll Radiol.

[21] Steyerberg EW, Vergouwe Y (2014). Towards better clinical prediction models: seven steps for development and an ABCD for validation. Eur Heart J.

[22] Daya D, Nazerali L, Frank GL (1992). Metastatic ovarian carcinoma of large intestinal origin simulating primary ovarian carcinoma. A clinicopathologic study of 25 cases. Am J Clin Pathol.

[23] Timmerman D, Planchamp F, Bourne T, Landolfo C, du Bois A, Chiva L (2021). ESGO/ISUOG/IOTA/ESGE Consensus Statement on preoperative diagnosis of ovarian tumors. Ultrasound Obstet Gynecol.

[24] Prat J (2017). Adhering to the 2014 WHO terminology on borderline ovarian tumors. Virchows Arch.

[25] Marko J, Marko KI, Pachigolla SL, Crothers BA, Mattu R, Wolfman DJ (2019). Mucinous neoplasms of the Ovary: radiologic-pathologic correlation. Radiographics.

[26] Kiyokawa T, Young RH, Scully RE (2006). Krukenberg tumors of the ovary: a clinicopathologic analysis of 120 cases with emphasis on their variable pathologic manifestations. Am J Surg Pathol.

[27] Khunamornpong S, Suprasert P, Na Chiangmai W, Siriaunkgul S (2006). Metastatic tumors to the ovaries: a study of 170 cases in northern Thailand. Int J Gynecol Cancer.

[28] Jordan SJ, Green AC, Whiteman DC, Webb PM (2007). Risk factors for benign, borderline and invasive mucinous ovarian tumors: epidemiological evidence of a neoplastic continuum?. Gynecol Oncol.

[29] Hunter SM, Gorringe KL, Christie M, Rowley SM, Bowtell DD, Campbell IG (2012). Pre-invasive ovarian mucinous tumors are characterized by CDKN2A and RAS pathway aberrations. Clin Cancer Res.

[30] Ikeuchi T, Koyama T, Tamai K, Fujimoto K, Mikami Y, Konishi I (2012). CT and MR features of struma ovarii. Abdom Imaging.

